# Research on serine hydroxymethyltransferase 2 mechanisms in malignant tumor development and progression

**DOI:** 10.7717/peerj.21570

**Published:** 2026-08-18

**Authors:** Mei Li, Ya Nan Xu, Xiulan Su, Jianxun Wen

**Affiliations:** 1 Center for Clinical Epidemiology Research, The Affiliated Hospital of Inner Mongolia Medical University, Hohhot, Inner Mongolia, China; 2 Public Service Center for Medical Research, Inner Mongolia Medical University, Hohhot, Inner Mongolia, China

**Keywords:** SHMT2, Tumor growth, Regulatory mechanism, Therapeutic target

## Abstract

SHMT2 (serine hydroxymethyltransferase 2) is a pivotal enzyme in cellular metabolism and is closely associated with the initiation and progression of malignant tumors, garnering significant attention in cancer research and therapy. Aberrant expression of SHMT2 has been observed in various cancers, correlating with tumor aggressiveness and patient prognosis. Functionally, SHMT2 participates in one-carbon metabolism, supplying methyl donors and biosynthetic precursors to support tumor cell proliferation. Additionally, it modulates redox homeostasis, promoting cancer cell survival and progression. SHMT2 also interacts with key signaling pathways, including JAK2/STAT3 and Akt/mTOR, thereby influencing tumor cell growth, invasion, and metastasis. Further elucidation of the molecular mechanisms by which SHMT2 contributes to tumorigenesis may provide critical insights for precision diagnosis, targeted therapy, and prognostic evaluationin oncology.

## Introduction

In the 1920s, Otto Warburg and colleagues observed that malignant tumor cells exhibit enhanced glycolytic activity for energy production even under aerobic conditions, a phenomenon now known as the “Warburg effect” (Warburg, 1956; Koppenol, Bounds & Dang, 2011; Dang & Semenza, 1999; Warburg, Wind & Negelein, 1927). This metabolic reprogramming is critical for sustaining rapid cancer cell proliferation, tumorigenesis, and evasion of cell death (Hanahan & Weinberg, 2011; Coelho, Fortunato & Carvalho, 2018; Hanahan, 2022; Vander Heiden & De Berardinis, 2017). One-carbon metabolism is a complex biochemical network mediated by folate-dependent reactions, comprising the folate and the methionine cycles. It supplies essential methyl donors and contributes to the synthesis and modification of macromolecules, including nucleic acids, proteins, and lipids, thereby playing a pivotal role in cellular proliferation, differentiation, and survival (Morscher et al., 2018; Tong et al., 2020). In cancer cells, one-carbon metabolism emerges as a key metabolic hub and a therapeutic target to support their accelerated growth. This metabolic adaptation not only provides energy and biosynthetic precursors for anabolic demands (Meiser & Vazquez, 2016; Appling, 1991) but also modulates signal transduction and epigenetic regulation, thereby driving tumor cell proliferation, invasion, and metastasis (Locasale, 2013; Ducker & Rabinowitz, 2017; Newman & Maddocks, 2017). The primary source of one-carbon units in cancer cells is derived from serine catabolism (Li & Ye, 2020).

Recent studies have identified SHMT2 as a pivotal enzyme in one-carbon metabolism, garnering significant attention in cancer research (gastric cancer, colorectal cancer, hepatocellular carcinoma, thyroid cancer, Kidney cancer, et al.). SHMT2 is primarily localized to the mitochondria and links the serine/glycine biosynthesis pathway with one-carbon metabolism. It catalyzes the reversible conversion of serine and tetrahydrofolate (THF) into glycine and 5,10-methylenetetrahydrofolate, a key reaction in one-carbon metabolism (Minton et al., 2018; Stover & Schirch, 1990). Therefore, SHMT2 expression may affect tumor cell metabolism and proliferation.

This review synthesizes recent literature on SHMT2, focusing on its structural and functional characteristics, expression profiles in malignant tumors, underlying molecular mechanisms, and emerging inhibitors. It aims to provide a theoretical foundation for SHMT2 in cancer prevention, diagnosis, and treatment. Scientific researchers interested in tumor research can gain interdisciplinary perspectives and entry points for collaboration on SHMT2 through this review. This review enables them to understand the academic value and potential clinical applications of SHMT2-targeted research, providing references for establishing scientific research projects, allocating medical resources, and informing related policy formulation.

### Structure and function of SHMT2

The SHMT2 gene is located at the chromosomal locus 12q13 on human chromosome 12 (Garrow et al., 1993). It encodes a protein with a molecular weight of approximately 55 kDa, which is primarily localized in mitochondria. The oligomeric state of SHMT2 dynamically shifts between dimers and tetramers, depending on its binding to the cofactor pyridoxal 5′-phosphate (PLP). SHMT2 shares approximately 66% amino acid sequence homology with its paralog, SHMT1, and together they constitute the SHMT family, which plays essential roles in cellular metabolism (Giardina et al., 2015; Stauffer, Plamann & Stauffer, 1981; Tramonti et al., 2021; Walden et al., 2019). Notably, an alternative short isoform, SHMT2*α*, has been identified. Unlike the canonical mitochondrial SHMT2, SHMT2*α* predominantly localizes to the cytoplasm and nucleus, where it functions as an inhibitory component of the BRISC (BRCC36 is a peptidase) complex (Walden et al., 2019; Zheng et al., 2013; Rabl et al., 2019). Both SHMT2 and SHMT2*α* can rescue glycine auxotrophy in GlyA-deficient cells, thereby restoring mitochondrial one-carbon metabolism (Anderson & Stover, 2009; Stover et al., 1997).

**Figure 1:**
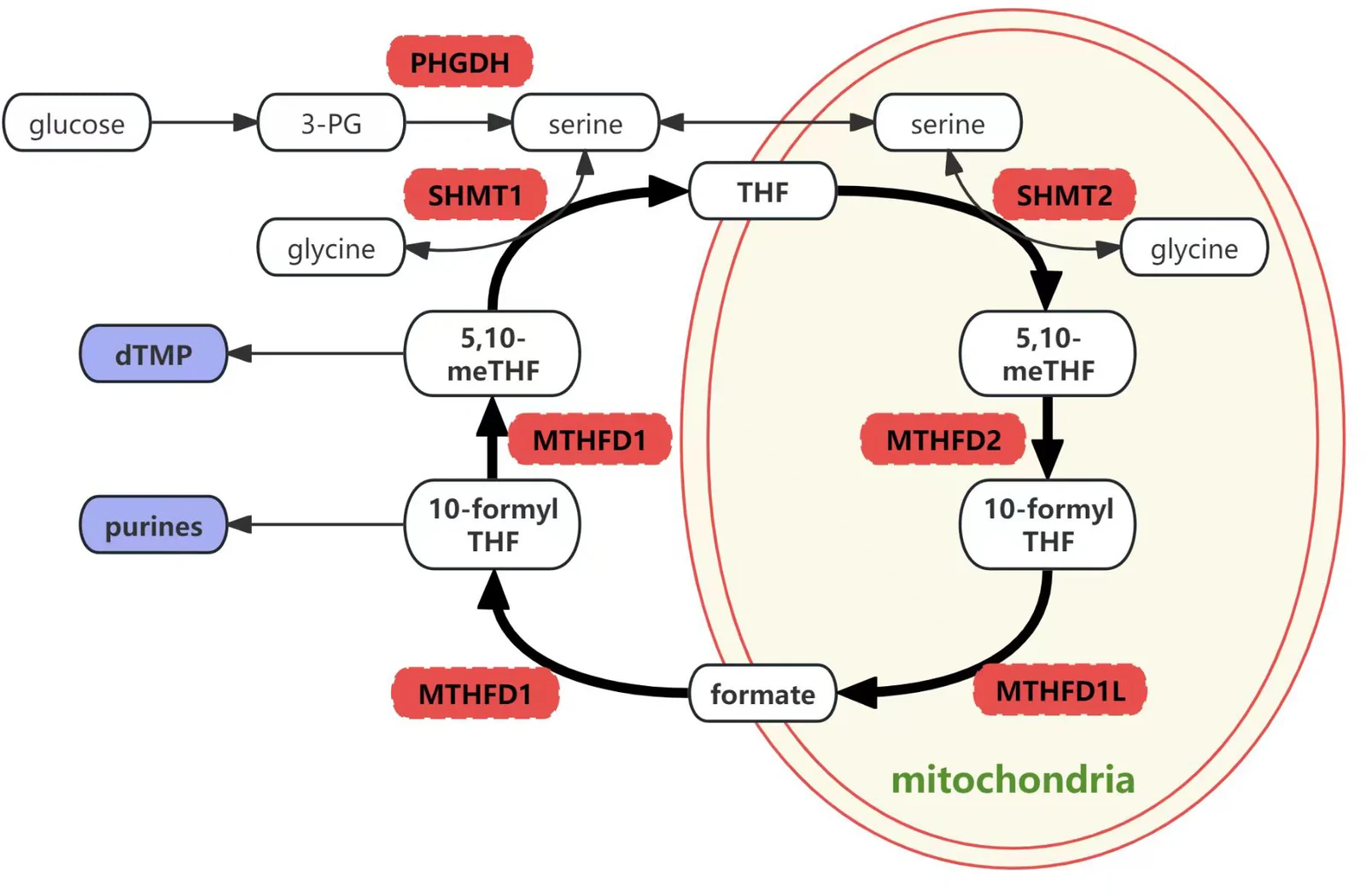
Schematic illustration of the role and mechanism of SHMT2 in serine/glycine synthesis pathways. SHMT2 is a pivotal enzyme in the serine/glycine synthesis pathway of single-carbon metabolism. SHMT2 contributes to mitochondrial 1C production, formate export, and NADPH balance (indirectly *via* folate cycle). SHMT2 catalyzes the conversion of serine to glycine and of tetrahydrofolate (THF) to 5,10-methylenetetrahydrofolate (5,10-CH2-THF). This metabolic pathway supplies essential precursors, glycine for protein synthesis and 5,10-CH2-THF for nucleotide biosynthesis, while also providing methyl groups for DNA synthesis and methylation, playing a vital role in maintaining cellular metabolic homeostasis and normal physiological functions (Kim, Nelson & Breaker, 2015).

SHMT2, a pivotal enzyme in the serine/glycine synthesis pathway and one-carbon metabolism, catalyzes the conversion of serine and tetrahydrofolate (THF) to glycine and 5,10-methylenetetrahydrofolate (5,10-CH2-THF) (Minton et al., 2018). This reaction not only provides glycine but also generates 5,10-CH2-THF, a crucial intermediate for various biosynthetic processes (Tong et al., 2020; Kim, Nelson & Breaker, 2015; Fu, Rife & Schirch, 2001) (Fig. 1). Furthermore, this metabolic pathway supplies essential precursors for protein, nucleotide, while also providing methyl groups for DNA synthesis and methylation, playing a vital role in maintaining cellular metabolic homeostasis and normal physiological functions (Mahmoud & Ali, 2019; Ducker et al., 2017). In tumor cells, the SHMT2-generated 5,10-CH2-THF contributes to mitochondrial NADPH production and maintains cellular redox balance (Kim et al., 2015; Ye et al., 2014; Fan et al., 2014). Additionally, SHMT2 can indirectly regulate tumor cell proliferation and survival by modulating intracellular glycine levels (Jain et al., 2012).

### Expression and functional significance of SHMT2 in cancers

SHMT2 is overexpressed in various malignant tumors and is associated with tumor occurrence and prognosis. In gastrointestinal cancers (including gastric, esophageal, and colorectal carcinomas), SHMT2 expression is significantly upregulated compared to adjacent non-tumor tissues, and high SHMT2 expression is an independent prognostic factor for adverse clinical outcomes (Liu et al., 2019; Miyo et al., 2017; Shi et al., 2019; Wang et al., 2023). Hepatocellular carcinoma cell lines (Huh-7, Hep3B, and HepG2) and intrahepatic cholangiocarcinoma tissues exhibit marked SHMT2 high expression. Patients with high SHMT2 expression have significantly shorter survival (Ning et al., 2018; Woo et al., 2016; Ji et al., 2019). Thyroid cancer (papillary and anaplastic subtypes) shows high expression of SHMT2, associated with TNM staging and high recurrence rate (Jin et al., 2021). Furthermore, SHMT2 and its co-expressed gene NDUFA4L2 are highly expressed in various renal carcinomas and predict worse overall survival in clear cell renal cell carcinoma patients (Wang et al., 2020). These studies have shown that SHMT2 is highly expressed in tumors and may serve as an important diagnostic/prognostic biomarker (Wu et al., 2017; Xie et al., 2022) (Table 1).

**Table 1:** The prognostic features of SHMT2 in different cancer types.

Cancer type	Data types	Methods	Expression	Prognostic
Gastric cancer	Clinical	Immunohistochemistry; qRT-PCR	High	Shortened overall survival time
Colorectal cancer	Clinical	Immunohistochemistry	High	Increased recurrence rate and shortened survival period
Hepatocellular carcinoma	Clinical	Immunohistochemistry; qRT-PCR	High	Shortened overall survival and increased tumor invasiveness
Thyroid cancer	Database	TCGA Database	High	TNM staging, increased recurrence rate
Kidney cancer	Cell lines and databases	Oncomine; Human Protein Atlas Database; ULCAN Database; qRT-PCR; Western blot	High	Shortened overall survival time

Inhibition of SHMT2 expression can significantly suppress cancer proliferation, migration and invasion both *in vitro* and *in vivo* (Liu et al., 2021; Chen et al., 2021). For instance, in colorectal cancer, downregulation of SHMT2 expression reduces cancer cell invasiveness and inhibits proliferation  (Cui et al., 2022). In tongue squamous cell carcinoma, downregulation of SHMT2 expression can significantly inhibit cancer cell proliferation, invasion, and migration, while inducing G1 phase arrest (Liao et al., 2021). Furthermore, reducing SHMT2 expression suppresses hepatocellular carcinoma cell proliferation and reduces tumorigenicity. In human tumor xenograft mouse models, SHMT2 knockdown inhibits Huh-7 cell tumor formation (Woo et al., 2016; Ji et al., 2019). These studies demonstrate the crucial role of SHMT2 in tumorigenesis and progression, and its potential as an effective therapeutic target for multiple cancer types.

### Mechanisms of SHMT2 in tumorigenesis

**Figure 2:**
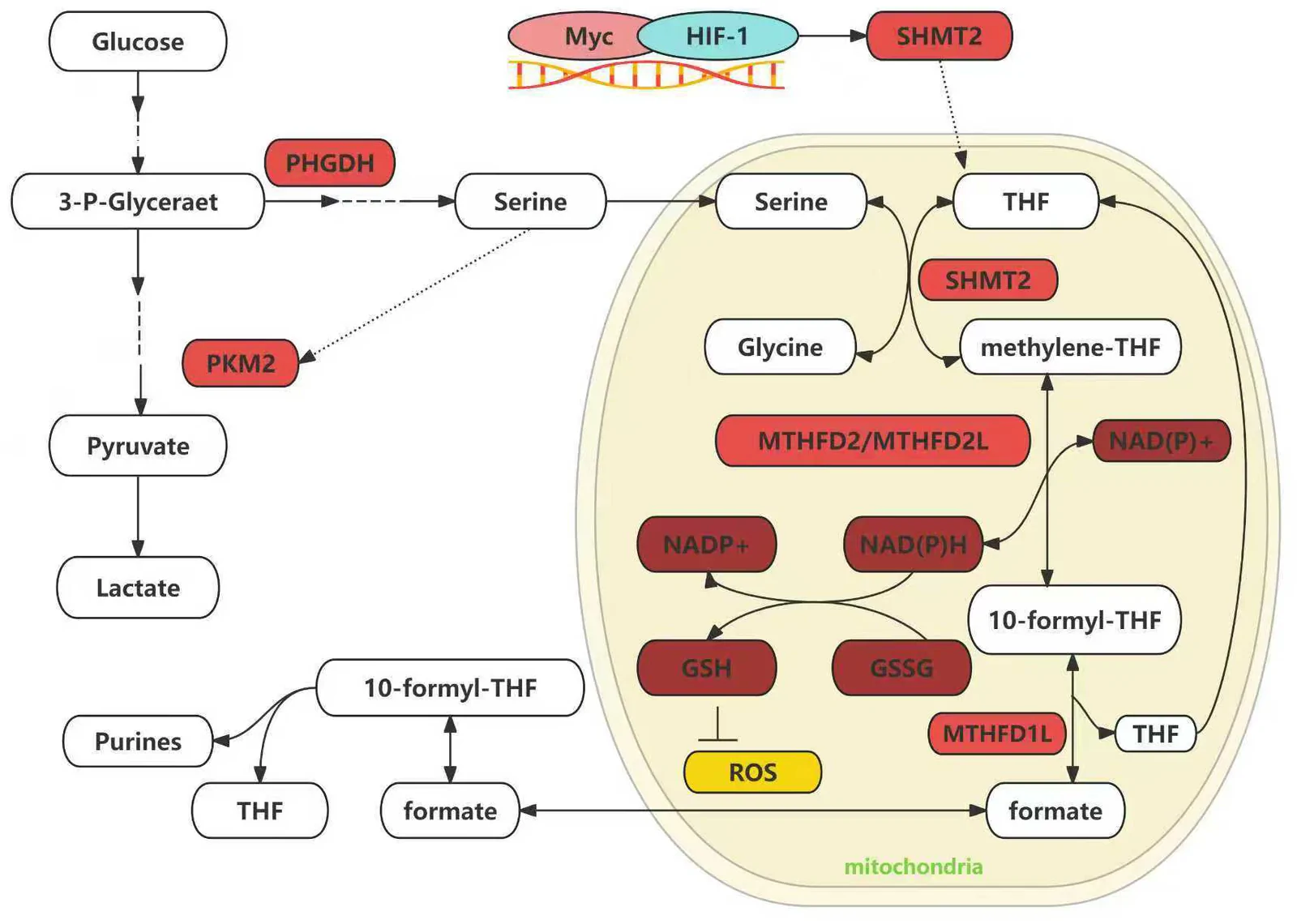
Schematic illustration of SHMT2 regulating redox mechanism in mitochondria. SHMT2 indirectly regulates NADPH and synthesizes glutathione *via* the mitochondrial folate cycle and MTHFD2/MTHFD1L-linked reactions. Under hypoxia or metabolic stress, MYC and HIF-1*α* co-regulate SHMT2. This regulation increases NADPH production from NADP+, reduces cellular reactive oxygen species (ROS), and improves tumor cell survival (Balamurugan, 2016).

SHMT2 regulates multiple signaling pathways to affect tumor occurrence, progression and metastasis. In one-carbon metabolism regulation, SHMT2 is involved in mitochondrial serine and one-carbon unit metabolism (Lucas et al., 2018). SHMT2 catalyzes the generation of methylenetetrahydrofolate, which subsequently produces NAD(P)H and formate, providing one-carbon units for the biosynthesis of nucleic acids, proteins, and lipids (Ducker et al., 2016), and promoting the rapid proliferation of breast cancer cells. Concurrently, SHMT2 overexpression enhances *de novo* purine synthesis (Lucas et al., 2018; Bernhardt et al., 2017; Zhang et al., 2016), thereby creating conditions essential for breast cancer cell proliferation (Li et al., 2020). Regarding redox balance modulation, the highly active metabolism in tumor cells generates substantial reactive oxygen species (ROS) (Guzy et al., 2005). The SHMT2-mediated serine metabolic pathway produces antioxidants, including NADPH, thereby maintaining a cellular reducing environment and promoting glutathione synthesis (Yang & Vousden, 2016). These antioxidants protect tumor cells from oxidative damage and facilitate their growth and survival (Ye et al., 2014). Studies have demonstrated SHMT2 as a transcriptional target of c-Myc (Nikiforov et al., 2002; Haggerty et al., 2003). C-Myc amplification upregulates SHMT2 expression in a HIF-1*α*-dependent manner, leading to increased NADPH production from NADP+, reduced cellular ROS levels, and enhanced tumor cell survival. SHMT2 has been experimentally verified to correlate with NADPH generation (Ye et al., 2014; Fan et al., 2014). The NADPH/NADP+ ratio directly regulates redox balance during hypoxia (Martínez-Reyes & Chandel, 2014), and HIF-1*α* can induce SHMT2 expression in hypoxic microenvironments (Fig. 2) (Ye et al., 2014; Balamurugan, 2016).

**Figure 3:**
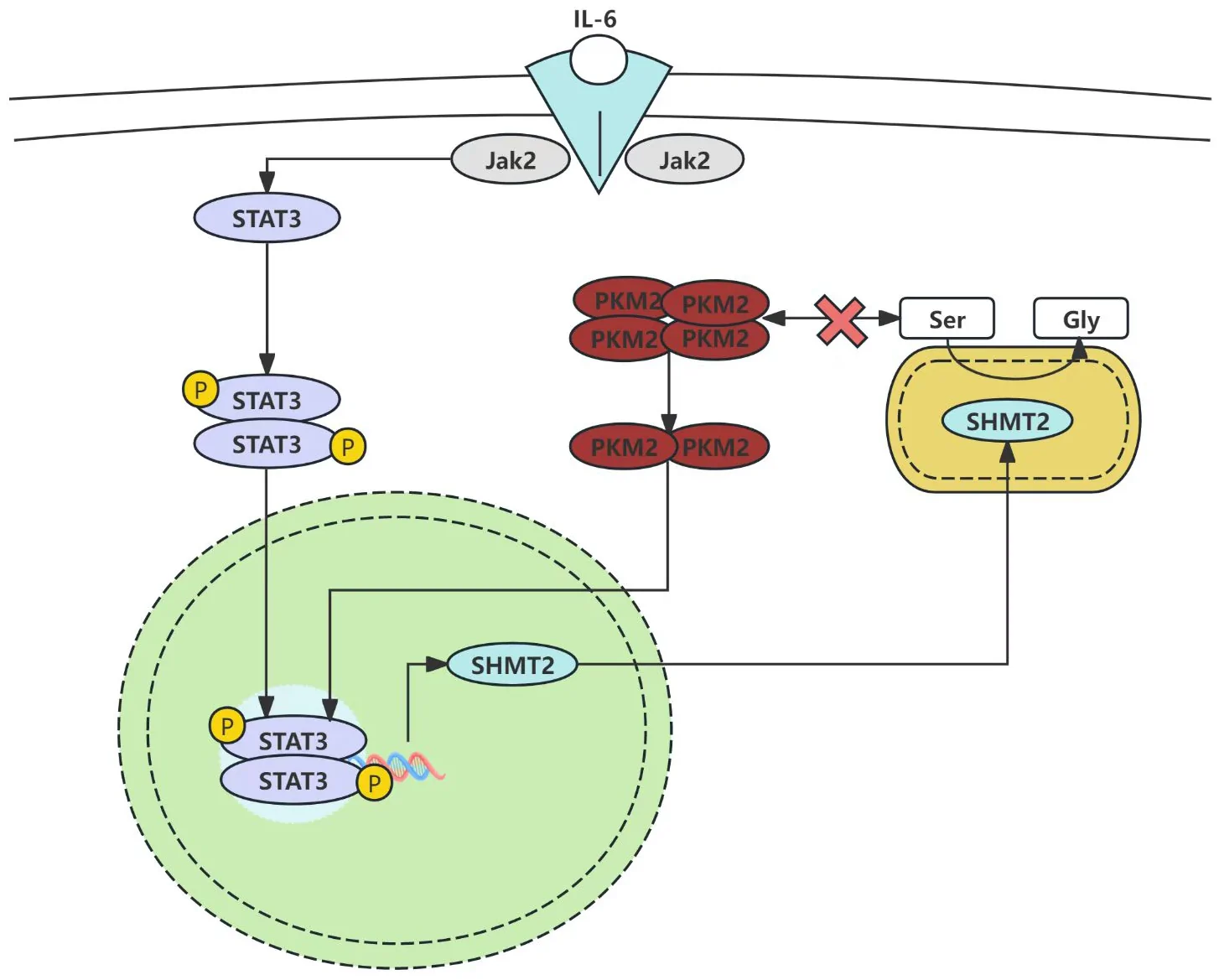
Schematic illustration of IL-6 activated STAT3/SHMT2/PKM2 signaling pathway. IL-6 upregulates SHMT2 expression by activating the JAK2/STAT3 pathway, associated with metabolic reprogramming and PKM2 nuclear signaling, and activates STAT3 in a non-canonical fashion, and promotes a transient shift toward anaerobic metabolism (Marrocco et al., 2019).

SHMT2, as a pivotal metabolic enzyme, not only regulates one-carbon metabolism and redox homeostasis but also interacts with multiple signaling pathways to modulate tumorigenesis and progression. In prostate cancer, SHMT2 demonstrates a close association with the STAT3 signaling pathway. IL-6 stimulation activates the JAK2/STAT3 pathway to upregulate SHMT2 expression, subsequently forming a STAT3/SHMT2/PKM2 feedback loop that reprograms tumor cell metabolism toward anaerobic glycolysis (Fig. 3) (Marrocco et al., 2019). In hepatocellular carcinoma, where the Akt/mTOR pathway plays crucial roles in cell growth, proliferation, survival, and metabolism, SHMT2 activation promotes hepatoma development (Wang et al., 2019). SHMT2 participates in the interaction between the transcription factor TCF4 and *β*-catenin, facilitating colorectal cancer growth and metastasis (Liu et al., 2021).

Post-translational modifications of SHMT2, including acetylation, succinylation, and delipoylation. In colorectal cancer, SIRT3-mediated SHMT2 deacetylation promotes tumor progression (Wei et al., 2018), SIRT5-induced SHMT2 desuccinylation enhances enzymatic activity, accelerating cellular metabolism and tumor growth (Yang et al., 2018). HDAC11-mediated SHMT2 delipoylation modulates type I interferon signaling, providing a theoretical basis for developing HDAC11 inhibitors in cancer therapy (Cao et al., 2019; Son et al., 2020; Deubzer et al., 2013). Notably, SHMT2 exhibits cancer type-specific mechanisms. For instance, in clear cell renal carcinoma, high SHMT2 expression correlates with tumor aggressiveness and poor prognosis (Wang et al., 2020).

In summary, the molecular mechanisms of SHMT2 in various cancers provide theoretical support for SHMT2-targeted cancer therapy.

### SHMT2-targeting inhibitors

As a key regulator of tumor cell metabolism, SHMT2 has emerged as a promising therapeutic target. Currently identified SHMT2 inhibitors are primarily included in Table 2.

**Table 2:** Types and targets of SHMT2 inhibitors.

Inhibitor	Core target(s)	Key activity indicator (core value)	Model system	Inhibitory effect	Types
SHIN1 (RZ-2994)	SHMT1 + SHMT2	Enzymatic IC_50_: SHMT1 = 5 nM; SHMT2 = 13 nM	Cell model; Animal model	SHMT1/2 inhibition	Tool compounds (experimental antifolate context, preclinical research)
SHIN2	SHMT1 + SHMT2	Prolongs survival of tumor-bearing mice	Cell model; Animal model	SHMT inhibition	Tool compounds (experimental antifolate context, preclinical research)
2.12	SHMT1-preferential	Enzymatic IC_50_: SHMT1 ≈0.3 μM	Cell model	SHMT1 inhibition	Tool compounds (experimental antifolate context, preclinical research)
AGF347	SHMT1/2 + Multiple Enzymes	Pancreatic cancer model tumor inhibition rate ≥ 50%	Cell model; Animal model	Antifolates (systemic blockers)	Tool compounds (experimental antifolate context, preclinical research)
NSC 127755 (DTB)	SHMT1 + SHMT2	Enzymatic IC_50_= 5 × 10^−8^ M	Cell model	Antifolates	Tool compounds (discontinued)
Lometrexol (LTX)	SHMT1 + SHMT2	36.5% residual SHMT1 activity at 100 μM	Cell model	Purine-antagonistic folate antagonist	Clinical Drug (Clinical Phase II, discontinued)
Pemetrexed (PTX)	SHMT1 + SHMT2 + Multiple Enzymes	Enzymatic Ki = 19.1 μM (SHMT1)	Cell model	Antifolates (systemic blockers)	Antifolate Drugs
5-Formyltetrahydrofolate (Leucovorin)	SHMT1 + SHMT2	Ki reaches nanomolar range after polyglutamylation	Cell model	Rescue folate	Antifolate Drugs
Metformin	SHMT2-preferential	Enzymatic Ki = 39 ± 6 mmol/L (SHMT2)	Cell model	Indirect metabolic modulators	Repurposed Drug
3-Bromopyruvate (3BP)	SHMT1-preferential	Enzymatic IC_50_= 30 μM (SHMT1)	Cell model; Animal model	Indirect metabolic modulators	Repurposed Drug

1. Pyrazolopyran and derivatives: The plant-derived pyrazolopyran scaffold represents a crucial chemical series for novel SHMT inhibitors. The small-molecule dual SHMT1/2 inhibitor SHIN1 depletes glycine to inhibit B-cell lymphoma growth, inhibits one-carbon metabolism in T-cell acute lymphoblastic leukemia cells, and inhibits diffuse large B-cell lymphoma (Ducker et al., 2017; Pikman et al., 2022). SHIN1 inhibits lung adenocarcinoma cell growth by suppressing SHMT2 (Han et al., 2024). SHIN2 improves survival in primary mouse T-cell acute lymphoblastic leukemia (T-ALL) models by reducing intracellular tetrahydrofolate levels, inhibiting SHMT expression, and inhibiting proliferation (García-Cañaveras et al., 2021). The compound 2.12, discovered in 2015, induces apoptosis in lung cancer cells, though its clinical application is limited by the high concentrations required for efficacy (Marani et al., 2016; Tramonti et al., 2018).

2. Folate analogs: The first SHMT inhibitor with demonstrated *in vivo* activity, AGF347, is a folate mimetic. Although exhibiting weaker SHMT2 inhibition than pyrazolopyrimidine compounds, AGF347 shows antitumor activity in both early- and advanced-stage pancreatic, lung, and colon cancers (Dekhne et al., 2019; Dekhne et al., 2020). NSC127755, the first SHMT inhibitor investigated, is an antifolate, but its development was discontinued due to severe adverse effects (Snell & Riches, 1989). Recent studies have shown that classical antifolates, including lometrexol and pemetrexed, also exhibit moderate inhibitory effects on both SHMT1 and SHMT2 (Daidone et al., 2011; Scaletti et al., 2019). Folinate compounds (*e.g.*, 5-formyl-THF) act as low-micromolar inhibitors of SHMT1/SHMT2 but are clinically unsuitable due to rapid *in vivo* conversion to other folate analogs (Chang et al., 2007; Stover & Schirch, 1991).

3. Other classes of inhibitors: Metformin disrupts SHMT2 tetramerization through a non-catalytic mechanism, inhibiting enzymatic activity (Tramonti et al., 2021). 3-Bromopyruvate (3BP), a novel antitumor agent, binds to the SHMT1 active site and inhibits SHMT1/SHMT2, and blocking one-carbon metabolism (Paiardini et al., 2016).

SHMT2 is a promising anticancer target, but no selective SHMT2 inhibitors have reached clinical approval or trials. This is due to poor drug developability, tumor resistance, limited research, and clinical barriers (Cuthbertson et al., 2021). Researchers should study SHMT2’s structure and its interactions with drugs to guide the design of safer, more effective SHMT2-targeted cancer drugs.

### Future perspectives

In tumor cells, folate-dependent one-carbon metabolism is crucial for cell proliferation. One-carbon (1C) units for purine and thymidine synthesis can be generated from serine by cytosolic or mitochondrial folate metabolism. The mitochondrial 1C pathway is consistently overexpressed in cancer. When the mitochondrial one-carbon pathway (with key enzymes such as SHMT2 and MTHFD2) is knocked out *via* CRISPR, the cytosolic one-carbon pathway triggers flux reversal through the depletion of 10-formyltetrahydrofolate (10-formyl-THF). Loss of the mitochondrial pathway, however, renders cells dependent on extracellular serine for 1C units and on extracellular glycine for glutathione. HCT-116 colon cancer xenografts lacking mitochondrial 1C pathway activity generate the 1C units required for growth by cytosolic serine catabolism. Loss of both pathways precludes xenograft formation  (Ducker et al., 2016). This compensatory mechanism provides new insights for tumor therapy. Dual-pathway therapy targeting one-carbon (1C) metabolism can use “mitochondrial SHMT2/MTHFD2 and cytosolic SHMT1” as the preferred combination. An alternative is “MTHFD2 and MTHFD1”. This approach has high tumor specificity and broad applicability across various tumor types. It shows clinical potential as a treatment strategy.

SHMT2 plays a pivotal role in the pathogenesis and progression of various malignant tumors. The expression level of SHMT2 is not only elevated and correlated with poor prognosis in lung cancer (Paone et al., 2014; De Nicola et al., 2015), but also associated with idiopathic pulmonary fibrosis (IPF) (Nigdelioglu et al., 2016). In addition, SHMT2 expression is associated with the survival of glioma cells in ischemic regions of gliomas (Kim et al., 2015). Moreover, SHMT2 sustains the proliferation of hematological malignancies (*e.g.*, T-cell acute lymphoblastic leukemia) by regulating one-carbon metabolism (Pikman et al., 2022; García-Cañaveras et al., 2021). Notably, SHMT2 is overexpressed in patients with osteoarthritis and contributes to its pathogenesis by modulating one-carbon unit metabolism (Song et al., 2015). SHMT2 exhibits potential as a core biomarker for clinical diagnosis, prognosis assessment, and molecularly targeted therapy. Through rigorous basic research, innovative drug development, and systematic clinical studies, studying SHMT2’s biological mechanisms will help develop more effective therapeutic strategies and provide new clues for future tumor-targeted therapy.

### Survey methodology

This study conducted a literature search *via* the PubMed and Web of Science databases (from 2000 to June 2025), and additionally supplemented with literature from search engines such as Google Scholar and CNKI as well as relevant academic journals; the search keywords included “SHMT2”, “tumor growth”, “regulatory mechanisms”, “therapeutic targets”, “SHMT2 inhibitors”, and others, with the search performed using combinations of Boolean operators: “SHMT2” AND “cancer”, “SHMT2” AND “carcinogenesis and progression”, “one-carbon metabolism regulation” AND “SHMT2”, “SHMT2” AND “prognostic marker”, “SHMT2” AND “therapeutic target”, “SHMT2 inhibitor” AND “preclinical study”.

Inclusion criteria: English-language original research articles and review papers related to the expression, mechanisms, and inhibitors of SHMT2 in tumors; exclusion criteria: conference abstracts, preprints, duplicate publications, and irrelevant literature. After deduplication and screening, 86 articles were included.

## Additional Information and Declarations

### Data Availability

The following information was supplied regarding data availability: This is a Literature Review.
